# Analysis of the cytotoxic and genotoxic effects in a population chronically exposed to coal mining residues

**DOI:** 10.1007/s11356-023-26136-9

**Published:** 2023-03-04

**Authors:** Grethel León-Mejía, Robinson Alvarez Rueda, Jose Pérez Pérez, Alvaro Miranda-Guevara, Ornella Fiorillo Moreno, Milton Quintana-Sosa, Cristiano Trindade, Yurina Sh De Moya, Martha Ruiz-Benitez, Yesit Bello Lemus, Ibeth Luna Rodríguez, Ludis Oliveros-Ortiz, Antonio Acosta-Hoyos, Leonardo C. Pacheco-Londoño, Amner Muñoz, Samuel P. Hernández-Rivera, Jesús Olívero-Verbel, Juliana da Silva, João Antonio Pêgas Henriques

**Affiliations:** 1 Centro de Investigaciones en Ciencias de La Vida (CICV), Universidad Simón Bolívar, Cra 53 Calle 64-51, 080002 Barranquilla, Colombia; 2 Grupo de Investigación en Química Y Biología, Universidad del Norte, Barranquilla, Colombia; 3 ALERT DHS Center of Excellence for Explosives Research, Department of Chemistry, University of Puerto Rico, Mayagüez, PR 00681 USA; 4 Environmental and Computational Chemistry Group, School of Pharmaceutical Sciences, Zaragocilla Campus, University of Cartagena, Cartagena, Colombia; 5 Laboratório de Genética Toxicológica, Universidade Luterana Do Brasil (ULBRA), Canoas-RS, Brazil; 6 Departamento de Biofísica, Centro de Biotecnologia, Universidade Federal Do Rio Grande Do Sul (UFRGS), Porto Alegre, RS Brazil; 7 Programa de Pós-Graduação Em Biotecnologia E Em Ciências Médicas, Universidade Do Vale Do Taquari - UNIVATES, Lajeado, RS Brazil

**Keywords:** Coal particles, DNA damage, Cytome, Oxidative damage, Cell death

## Abstract

During coal mining activities, many compounds are released into the environment that can negatively impact human health. Particulate matter, polycyclic aromatic hydrocarbons (PAHs), metals, and oxides are part of the complex mixture that can affect nearby populations. Therefore, we designed this study to evaluate the potential cytotoxic and genotoxic effects in individuals chronically exposed to coal residues from peripheral blood lymphocytes and buccal cells. We recruited 150 individuals who lived more than 20 years in La Loma-Colombia and 120 control individuals from the city of Barranquilla without a history of exposure to coal mining. In the cytokinesis-block micronucleus cytome (CBMN-Cyt) assay, significant differences in the frequency of micronucleus (MN), nucleoplasmic bridge (NPB), nuclear bud (NBUD), and apoptotic cells (APOP) were observed between the two groups. In the buccal micronucleus cytome (BM-Cyt) assay, a significant formation of NBUD, karyorrhexis (KRX), karyolysis (KRL), condensed chromatin (CC), and binucleated (BN) cells was observed in the exposed group. Considering the characteristics of the study group, a significant correlation for CBMN-Cyt was found between NBUD and vitamin consumption, between MN or APOP and meat consumption, and between MN and age. Moreover, a significant correlation for BM-Cyt was found between KRL and vitamin consumption or age, and BN versus alcohol consumption. Using Raman spectroscopy, a significant increase in the concentration of DNA/RNA bases, creatinine, polysaccharides, and fatty acids was detected in the urine of individuals exposed to coal mining compared to the control group. These results contribute to the discussion on the effects of coal mining on nearby populations and the development of diseases due to chronic exposure to these residues.

## Introduction

Coal is a mineral and a very useful resource throughout the world, particularly thermal-type coal, which is used in power plants to generate electricity (Lin et al. 2019). In addition to power generation, gasification, and coke production, coal is used in the production of benzol, oils, and tar and can be used as a substitute for oil through liquefaction (Hendryx et al. 2020; Souza et al. 2021).

During coal mining activities, large amounts of particles, ash, metals, oxides, and PAHs are released into the environment (Hendryx et al. 2020; Finkelman et al. 2002). Coal particles are chemically complex and can trigger the activation of macrophages, epithelial cells, and fibroblasts; the release of reactive oxygen species (ROS); and the expression of cytokines (Gulumian et al. 2006). Proinflammatory cytokines play an important role in the lung inflammatory response and oxidative stress in individuals exposed to occupational pollutants (Berumen-Rodríguez et al. 2022; Díaz de León-Martínez et al. 2022; Zhou et al. 2014). In some populations, exposure to compounds derived from coal mining begins before birth and continues throughout their life cycle (Barn et al. 2019). Many compounds generated during coal mining have been related to different diseases, such as asthma, bronchitis, emphysema, pneumoconiosis, and different types of cancer (Gulumian et al. 2006; Lin et al. 2019; Prasad et al. 2021).

Currently, the use of biomarkers is fundamental in assessing the effects and extent of damage at the cellular or chromosomal level in human populations exposed to different genotoxic agents (Kapeleka et al. 2021). One of the most widely used assays is the cytokinesis-block micronucleus cytome (CBMN-Cyt) assay of peripheral blood lymphocytes because it is a promising approach for measuring DNA damage, cytostatic effects, and cytotoxicity (Fenech 2007). DNA damage events are specifically measured in once-divided binucleated (BN) cells and include the following: (1) micronucleus (MN) measurement, which is a biomarker of chromosome breakage and/or complete chromosome loss; (2) measurement of nucleoplasmic bridges (NPBs), which are biomarkers of poor DNA repair or fusion of the ends of telomeres; and (3) measurement of nuclear buds (NBUDs), which are considered biomarkers of the removal of amplified DNA and/or DNA repair complexes (Fenech 2007).

The buccal micronucleus cytome (BM-Cyt) assay has also become one of the preferred assays for human biomonitoring studies because it has advantages over other methodologies considering that it is a noninvasive method performed using epithelial cells exfoliated from the buccal mucosa and does not require cell culture and allows analysis of cytotoxicity, DNA damage, and defects in cytokinesis (Bolognesi et al. 2015; Thomas et al. 2009).

Therefore, this study was designed to evaluate the potential cytotoxic and genotoxic effects of coal residues on peripheral blood lymphocytes and buccal cells in individuals chronically exposed to them. The results obtained in this study will contribute to the discussion on the effects of coal mining activities, with which the implementation of educational programs and better public health strategies and the surveillance of these populations are expected.

## Materials and methods

### Subjects and sampling

**Fig. 1:**
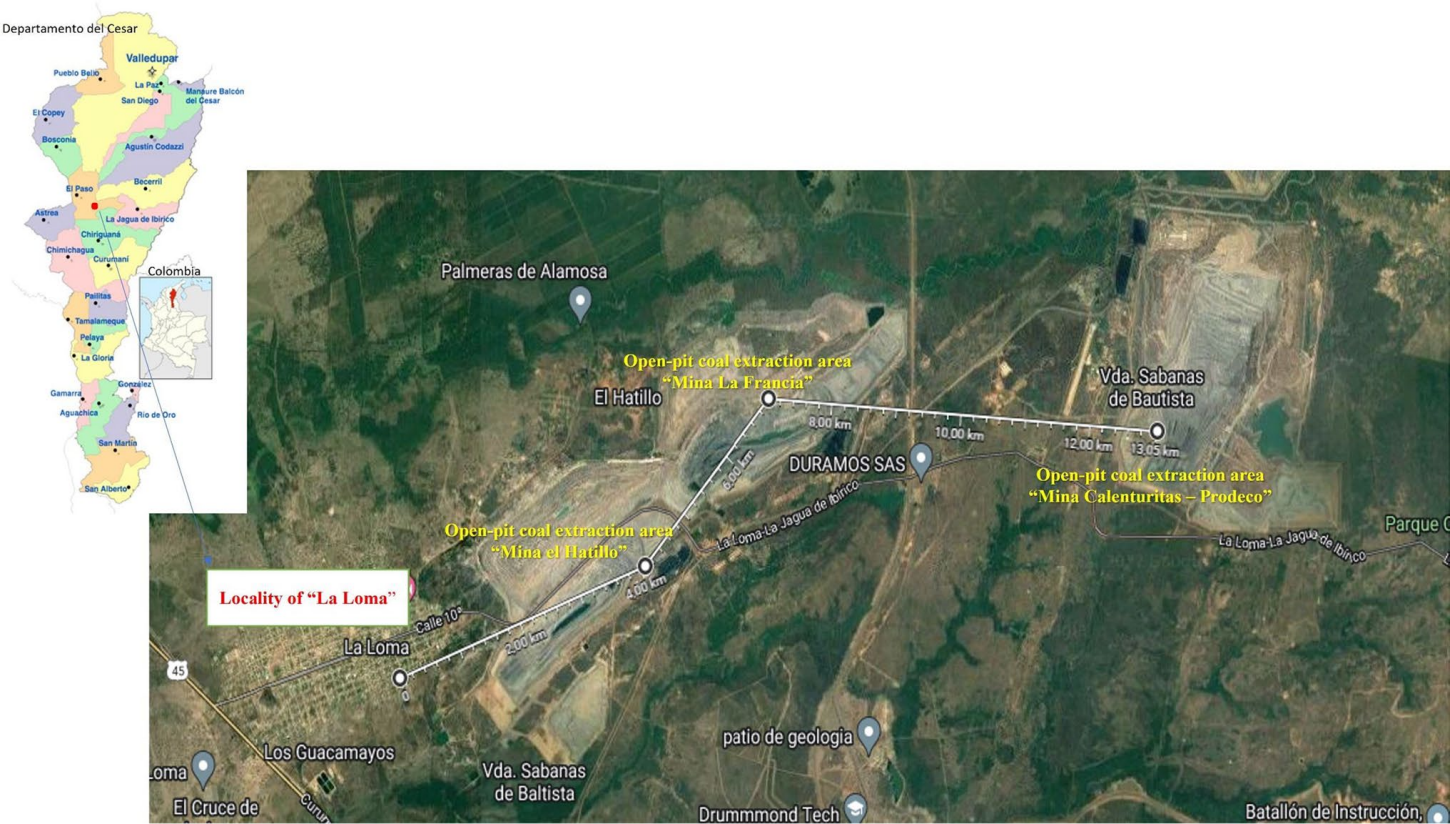
Distance from the locality of “La Loma” to the areas of coal mining extraction (Google maps and Gobernación del Cesar, 2023)

The study subjects were individuals who lived for > 10 years in the mining region of “La Loma,” Department of Cesar-Colombia (South America). The locality of “La Loma” is located 2 km from the first extraction area. Around “La Loma,” there are three open-pit coal extraction areas (Fig. 1). This study included 150 individuals (89 women and 61 men) from “La Loma” and 120 control individuals (70 women and 50 men) from the city of Barranquilla without a history of exposure to coal mining. These two groups were matched for age (± 2 years). To obtain information on lifestyle habits and confounding factors, the subjects of this study answered a questionnaire that included information on health status, cancer history, other chronic diseases, nutrition, smoking habit, medication intake, frequency of alcohol consumption (total number of drinks and most consumed alcoholic beverages), occupation, previous exposure to X-rays, and treatment with known carcinogens. The exposed group was selected according to the following inclusion criteria: voluntary acceptance and have been born or have at least 10 years of living in the La Loma mining region. The exclusion criteria for the exposed and control groups were exposure to other risk factors (i.e., genotoxins), medical treatment up to 3 months before sampling, X-ray exposure up to 1 year before sampling, diagnosis of cancer, chemotherapy/radiotherapy, and intake of therapeutic drugs known to be mutagenic.

All participants of this study were informed about the objectives and methodologies that will be used, and they provided informed consent for the voluntary acceptance in this work. This study was approved by the Ethics Committee of the Universidad Simón Bolívar (CIE-USB-CE-0233–00). The information obtained from the study participants was organized in databases. All identifying pieces of information were stored at the Institute of Life Sciences of the Universidad Simón Bolívar in Barranquilla (Departamento del Atlántico).

### Sample collection

After the subjects provided informed consent, 5 mL of blood was obtained by venipuncture in heparin tubes. For buccal cell samples, each participant rinsed their mouth with water before the samples were taken. The exfoliated buccal mucosa cells were collected using a cytobrush by gently scraping the mucosa of the inner lining of both the cheeks. All sample tubes were coded and stored in an upright position in ice and in the dark during transportation to the laboratory where the samples were immediately processed upon arrival.

### CBMN-Cyt assay

For the analysis of lymphocyte cytome biomarkers, two peripheral blood cultures were performed per individual. Each culture contained 0.5 mL of whole blood in 4.5 mL of medium (Roswell Park Memorial Institute 1640) supplemented with 10% fetal bovine serum, 1% L-glutamine, and 1% antibiotics (streptomycin–penicillin). Lymphocytes were stimulated with 2% phytohemagglutinin and incubated at 37 °C for 72 h. Forty-four hours after the start of the cultures, 0.2 mL of cytochalasin B at a concentration of 4.5 ug/mL was added to each culture to inhibit cytokinesis and obtain BN cells (first cycle of cell division). The cells were harvested at 72 h by centrifugation and treated with hypotonic KCl solution (0.075 M) and fixed with Carnoy’s fixative. Cytogenetic preparations were coded (double-blind) and stained with 10% Giemsa. For the analysis, 1000 BN cells were analyzed under a light microscope. For this, 500 cells per slide were analyzed and classified to determine the NDI, which is a biomarker of cytostasis and measures the proliferative status of cells.

**Fig. 2:**
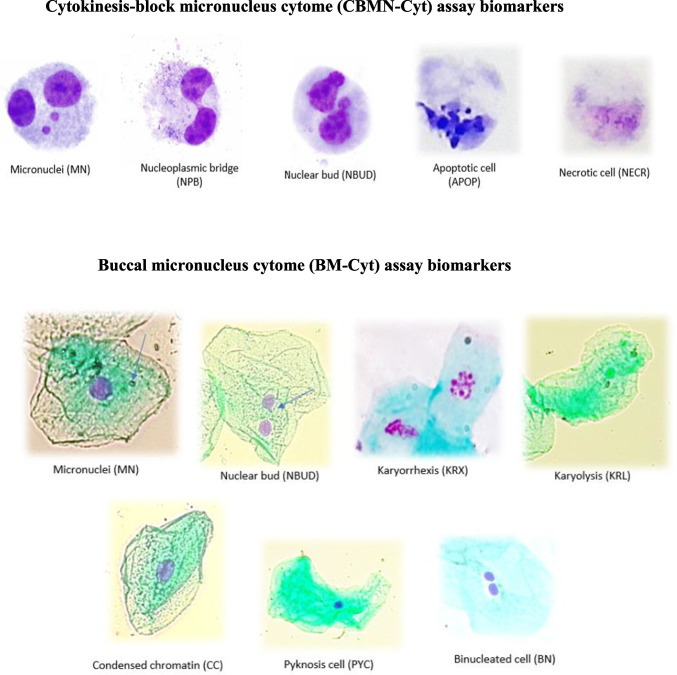
Cytokinesis-block micronucleus (CBMN-Cyt) and buccal micronucleus cytome (BMCyt) assay biomarkers analyzed

The NDI was calculated using the following formula:

NDI = (M1 + 2M2 + 3M3 + 4M4)/N, where M1–M4 represents the number of cells with 1–4 nuclei, and N is the total number of viable cells measured (excluding necrotic and apoptotic cells). Cytotoxic effects were evaluated using the frequency of NECR and APOP. For which, 500 cells were randomly measured. DNA damage biomarkers, such as the frequency of MN, NPB, and NBUD, were analyzed in 1000 binucleated cells per slide (Fig. 2). All biomarker analyses of the CBMN-Cyt assay were performed following the recommendations of Fenech (2007).

### BM-Cyt assay

The samples of exfoliated cells from the mouth were mixed with 5-mL cold saline (0.9% (w/v) aqueous NaCl). Then, they were centrifuged at 1500 rpm for 10 min, and the sedimented buccal cells were washed twice with saline and once with Carnoy’s fixative (methanol and glacial acetic acid, 3:1) under the same centrifugation conditions. To perform the analyses, 100 µL of cell suspension was dropped onto a microscope slide and immediately spread using a pipette tip. Fixed cells were hydrolyzed and stained using the Feulgen method according to the recommendations of Thomas et al. (2009). For the analysis of BM-Cyt biomarkers, two slides per individual were created, and 1000 cells per slide were counted. Biomarkers of DNA damage (MN and NBUD), cell death (CC, PYC, KRX, and KRL), proliferative potential (basal cell frequency), and cytokinesis defect (BN cells) (Fig. 2) were evaluated according to the recommendations of Thomas et al. (2009).

### Surface-enhanced Raman scattering spectroscopy

Surface-enhanced Raman scattering (SERS) was performed by the interaction of gold nanoparticles (NP-Au) with the urine metabolites. Acquisition of Raman spectra was measured in a Wasatch Photonics Raman spectrometer, with a work range 270–2000 cm^−1^ to 785 nm excitation line. For this, 5 uL of NP-Au were deposited on a gold sheet, followed by 5 uL of sample on a droplet of NP-Au and a mixture using the same tip. Next, the Raman spectra was collected for an acquisition time of 1 s and a power laser of 20 mW.

The NP-Au were synthesized by mixing 300 µL of a 20 mM AuCl3 solution, 65 µL 2% w/v sodium citrate, and 640 µL of 18 MOhm deionized water. The sizes of NP-Au were measure in a Zetasizer Lab Dynamic Light Scattering instrument (Malvern Panalytical), the size was 51 ± (− 8, + 11) nm. NP-Au shown a plasmon resonance of 536 nm, this was measure in a UV–VIS spectrometer CLARIOstar Plus.

The spectral were preprocessed by second-order polynomial baseline removal (Lee et al. 2018) following Standard Normal Variate Scaling (SNV) (Lau et al. 2012). This was done in order to normalize the Raman signal and that the proportions of the metabolites were relative. The preprocessing was carried out using the PLS Toolbox version 7.5.2 (Eigenvector Research Inc., Wenatchee, WA, USA) with the MATLAB R2016b version 7 platform (MathWorks, Natick, MA, USA).

### Statistical analysis

To test the normality of the variables, the Kolmogorov–Smirnov test was performed. Statistical analysis also included an analysis of differences in the biomarkers using the nonparametric Mann–Whitney *U*-test, *t*-student test, and Spearman’s correlation analysis to analyze the influence of age, lifestyle variables, and family history of cancer, as well as the correlations between different cell parameters. The critical level for the rejection of the null hypothesis was set at 5%. All these analyses were performed using GraphPad Prism 5.0.

## Results

Table 1 shows the main characteristics of the study groups. The mean age of the individuals in the control group was 38.5 ± 11.8 years (range, 13–71 years), and that of the individuals in the exposed group was 40.3 ± 16.2 years (range, 12–74 years). The average exposure time was 20.8 years. Regarding the life habits of the study population, 60% of the individuals in the control group and 55% of those in the exposed group consumed alcohol (defined as drinking > 3 bottles of beer per day or drinking in excess once a week).

**Table 1:** Main characteristics of the control and exposed groups

Population characteristics	Control	Exposed
Number of individuals	120	150
Males	50	61
Females	70	89
Age, years (mean ± SD)	38.5 ± 11.8	40.3 ± 16.2
Alcohol consumption*		
Yes	60.0%	55.0%
No	40.0%	45.0%
Vitamins consumption		
Yes	60.0%	18.0%
No	40.0%	82.0%
Red meat consumption		
Yes	93.0%	94.0%
No	7.0%	6.0%
White meat consumption		
Yes	90.0%	40.0%
No	10.0%	60.0%
Consumption of vegetables		
Yes	84.0%	58.0%
No	16.0%	42.0%
Consumption of fruits		
Yes	93.0%	57.0%
No	7.0%	43.0%
Family history of cancer		
Yes	22.0%	42.0%
No	78.0%	58.0%

*SD*, standard deviation

^*^Drink more than three beers/day or in excess once a week

Moreover, the exposed group had low consumption of vitamins (82% of the individuals in this group did not consume vitamins) compared with the control group (40% of the individuals in this group did not consume vitamins). A high consumption of red meat was also shown, and more than 50% of the individuals in the exposed group consume fruits and vegetables. The exposed group had lower consumption of white meat (i.e., chicken and fish) than the control group (40% vs. 90%). Regarding family history of cancer, the percentage of individuals with a family history of cancer was low in both the control group (22%) and exposed (42%) groups.

Table 2 shows the CBMN-Cyt and BM-Cyt assay biomarkers in the control and exposed groups. Regarding CBMN-Cyt biomarkers, the results indicate significant differences in the frequency of MN, NPB, NBUD, and APOP between the two groups (Mann–Whitney *U*-test, *p* < 0.0001). Regarding BM-Cyt biomarkers, a significant formation of NBUD, KRX, KRL, CC, and BN was observed in the exposed group compared to the control group (Mann–Whitney *U*-test, *p* < 0.0001).

**Table 2:** Cytokinesis-block micronucleus cytome (CBMN Cyt) and buccal micronucleus cytome assay (BM-Cyt) assay biomarkers in the control and exposed group

Parameters	Control (*n* = 120)	Exposed (*n* = 150)
CBMN-Cyt		
MN	4.1 ± 2.6	8.8 ± 6.4***
NPB	2.0 ± 1.1	4.2 ± 2.2***
NBUD	2.9 ± 1.0	3.8 ± 2.7**
APOP	8.6 ± 4.3	10.3 ± 8.2*
NECR	6.6 ± 1.4	7.0 ± 4.1
NDI	1.99 ± 0.05	1.98 ± 0.06
BM-Cyt		
MN	1.7 ± 1.4	2.0 ± 1.4
NBUD	1.0 ± 0.8	2.0 ± 0.1*
KRX	20.7 ± 10.9	32.2 ± 10.6***
KRL	33.2 ± 6.5	58.3 ± 29.7***
CC	21.0 ± 7.7	37.8 ± 12.9***
PYC	2.8 ± 1.1	3.1 ± 2.0
BN	10.5 ± 4.9	30.2 ± 15.3***

*MN*, micronuclei; *NPB*, nucleoplasmic bridge; *NBUD*, nuclear bud; *APOP*, apoptotic cells; *NECR*, necrotic cells; *NDI*, nuclear division index, *KRX*, karyorrhexis; *KRL*, karyolysis; *CC*, condensed chromatin; *PYC*, pyknosis; *BN*, binucleated. Data are expressed as means ± standard deviations. *Significant difference in relation to the control group; Mann–Whitney *U*-test,* p* < 0.0001

In this study, in the exposed group, 89 women and 61 men were included. Among the analyzed biomarkers, only CBMN-Cyt-MN had significant values in women compared with those in men. The MN frequency was 9.4 ± 3.8 for women and 8.2 ± 5.5 for men (*p* < 0.05, data not shown).

**Fig. 3:**
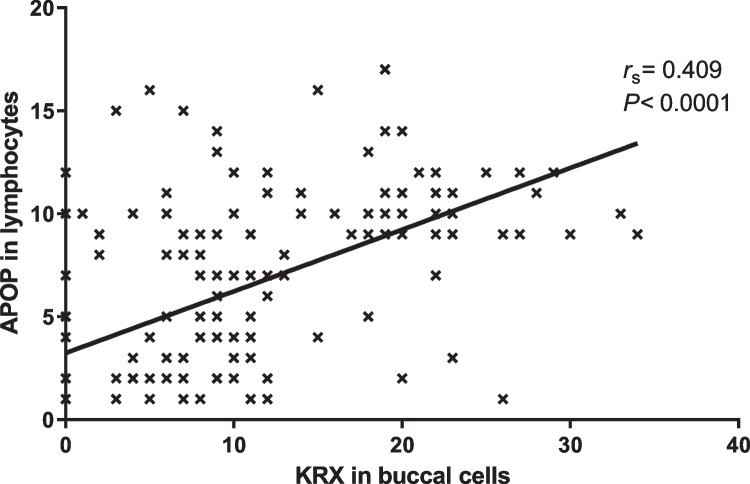
Nonparametric Spearman’s correlation analysis between apoptosis (APOP) frequency in lymphocytes and karyorrhexis (KRX) in buccal cells

Figure 3 shows the results of the nonparametric Spearman’s correlation analysis for the only significant correlation found (between APOP frequency in lymphocytes and KRX in buccal cells; *p* < 0.0001, *r*_*s*_ 0.409).

Table 3 shows the results of Spearman’s correlation analysis of CBMN-Cyt and BM-Cyt assay biomarkers in the exposed group in relation to the characteristics of the study group. Significant correlations were found between CBMN-Cyt-NBUD and vitamin consumption (*p* = 0.020), between CBMN-Cyt-MN and meat consumption (*p* = 0.013), CBMN-Cyt-MN and age (*p* = 0.025), and between CBMN-Cyt-APOP and meat consumption (*p* = 0.035). Regarding BM-Cyt biomarkers, significant correlations were found between BM-Cyt-KRL and vitamin consumption (*p* = 0.001), BM-Cyt-KRL and age (*p* = 0.006) and between BM-Cyt-BN and alcohol consumption (*p* = 0.033).

**Table 3:** Spearman’s correlation analysis of cytokinesis-block micronucleus cytome (CBMN-Cyt) and buccal micronucleus cytome (BM-Cyt) assay biomarkers in the exposed group in relation to the characteristics of the study group. Analysis performed using the average of each biomarker

Correlations	*r*_s_	*p*
CBMN-Cyt-NBUD versus vitamins consumption CBMN-Cyt-MN versus meat consumption	0.277	0.020
	0.252	0.013
CBMN-Cyt-MN versus age	0.215	0.025
CBMN-Cyt-APOP versus meat consumption	0.110	0.035
BMCyt-KRL versus age	0.174	0.006
BMCyt-KRL versus vitamins consumption	0.155	0.001
BMCyt-BN versus alcohol consumption	0.136	0.033

*NBUD*, nuclear bud; *MN*, micronuclei; *APOP*, apoptotic cells; *KRL*, karyolysis; *BN*, binucleated

**Fig. 4:**
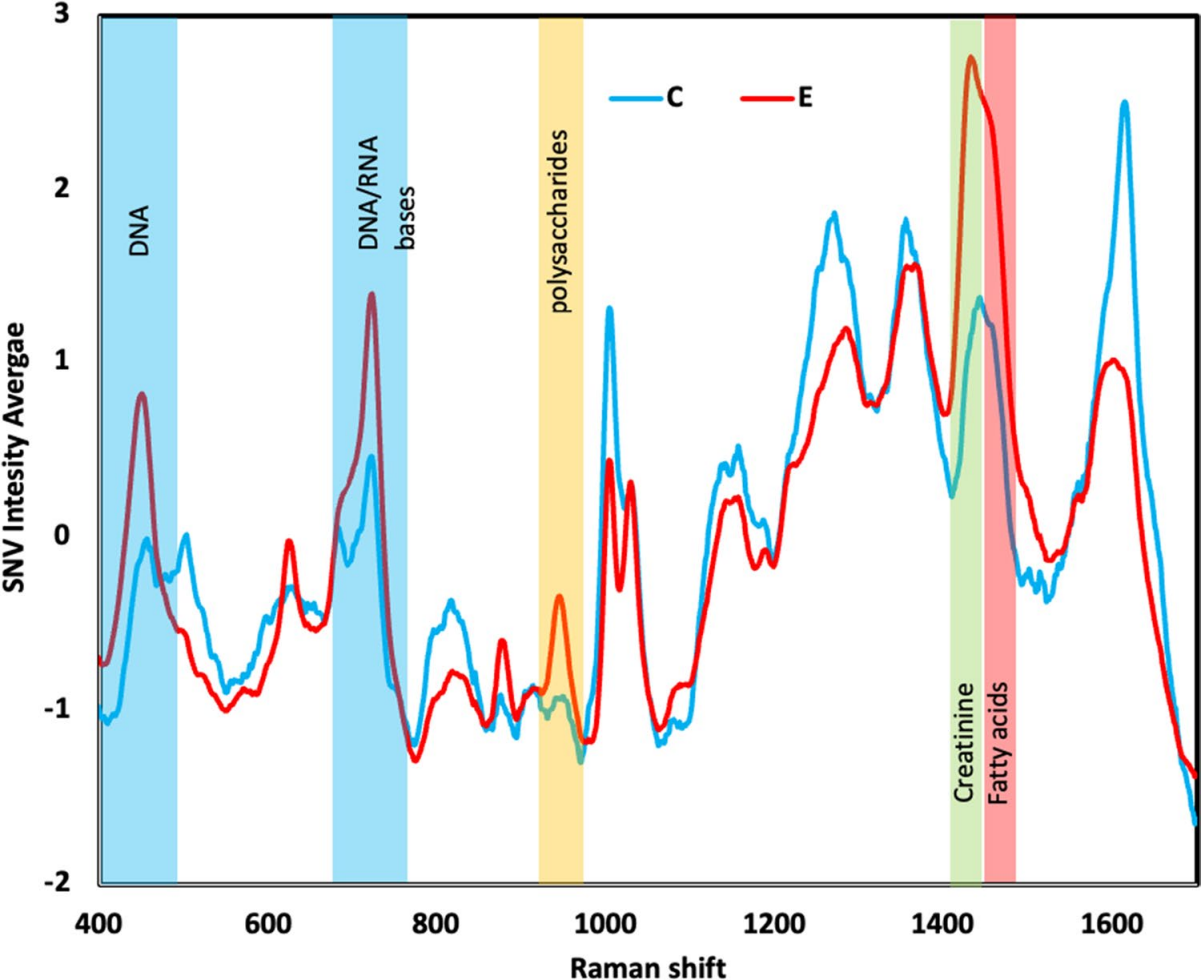
Average of normalized Raman spectra by SNV for sample of urine of individuals exposed to coal mining (E) and controls (C) and the band assignment

**Fig. 5:**
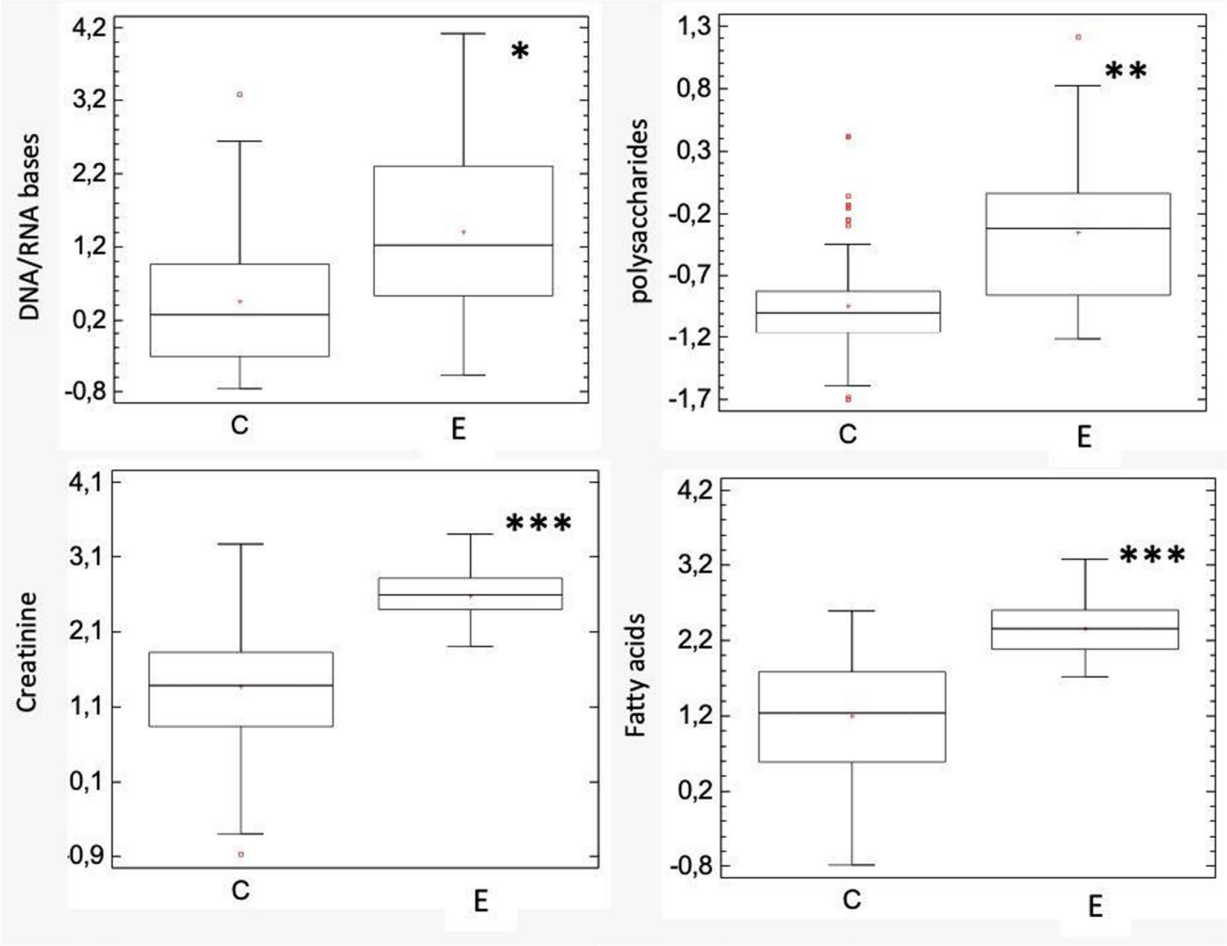
Box plot for the metabolite bands that showed an increase in the individuals exposed to coal mining (E, N = 150) vs. controls (C, N = 120). *Statistically significant difference in relation to control group, statistical test t-student (*p* < 0.0001)

Figure 4 shows the average of normalized Raman spectra by SNV between urine samples of individuals exposed to coal mining (E) and controls (C). We found 5 bands where the relative intensity for E is increased with respect to C, with signals at 449 cm^−1^, 724 cm^−1^, 945 cm^−1^, 1442 cm^−1^, and 1458 cm^−1^, where we assigned these bands based on Raman analysis reported in the literature. In Fig. 4, these bands are highlighted in colors, the DNA bands (449 cm^−1^, 724 cm^−1^) (Lin et al. 2020; Talari et al. 2015) are highlighted in blue, for polysaccharide (945 cm^−1^) (Shetty et al. 2006) in yellow, creatinine (1442 cm^−1^) (Lin et al. 2020) in green and fatty acids (1458 cm^−1^) (Li et al. 2016) in red. A statistical significance test was performed for each type of metabolite (*p*-value < 0.0001) and a box plot for each metabolite was plotted to observe the differences in distributions between the two groups (Fig. 5).

## Discussion

Coal mining has a negative impact on human health and causes irreparable damage to natural ecosystems (Feng et al. 2020). Studying the cytotoxic and genotoxic effects of coal mining on exposed populations should be a relevant issue for understanding the agents released into the environment and their consequences on organisms (León et al. 2007; León-Mejía et al. 2011; Rohr et al. 2013; Zocche et al. 2013).

In this study, the results showed a significant increase in MN, NPB, and NBUD in the exposed group compared to the control group. Several studies have demonstrated the genotoxic risk due to chronic exposure to coal mining (Rohr et al. 2013; Sinitsky et al. 2017; Souza et al. 2021). In our findings, the frequency of APOP was also significant. When DNA damage is tolerable, it can lead to different responses, such as cell cycle arrest and DNA repair; however, when the damage is excessive or irreparable, it can lead to cell death to counteract the damage caused by carcinogens (Jarvis et al. 2014; Matt and Hofmann 2016). Regarding the NDI biomarker, which measures cytostasis and the proliferative state of the viable cell fraction, no significant differences were observed between the exposed and control groups (León-Mejía et al. 2019; Quintana-Sosa et al. 2021).

Moreover, we detected a significant increase in the concentration of DNA/RNA bases, creatinine, polysaccharides, and fatty acids in the urine of the population exposed to coal mining compared to the control group. These alterations can be attributed to the complex mixture released during coal mining activities to which the inhabitants of that region are continuously exposed (León-Mejía et al. 2014). Compounds such as metals (Angelé-Martínez et al. 2014), PAHs (Lin et al. 2022), and oxides (Liou et al. 2017) have the capacity to induce the formation of ROS; in addition, the inhalation of these particles can induce inflammatory effects in the lung, which leads to oxidative damage in macromolecules such as proteins (de Oliveira Alves et al. 2020), lipids (Niu et al. 2020) and nucleic acids (Niu et al. 2020). The increase in biomarkers detected may be related to the oxidation of these macromolecules which leads to cell death and its elimination can be evidenced in the urine.

Regarding sexual differences, in the CBMN-Cyt assay, women had a significantly higher frequency of MN than men. Several studies corroborate the high frequencies of MN in women due to multiple factors, such as X chromosome inactivation. Women have two copies of the chromosome compared with men who have only one copy, and it is probably lost as MN in relation to other chromosomes (Donmez-Altuntas and Bitgen 2012; Fenech and Bonassi 2011; Gajski et al. 2018). It can also be due to and hormonal effects which contribute to further DNA oxidative damage and cancer development (Hsu et al. 2010; Zhang et al. 2022).

In the BM-Cyt assay, the results revealed no significant difference in MN formation between the exposed and control groups. The frequency of the formation of micronuclei is lower in the oral mucosa than in peripheral blood (Bonassi et al. 2011; Holland et al. 2008). However, a significant increase in NBUD formation was found. NBUD formation indicates gene amplification, and this has great biological relevance if we consider that the number of copies of a part of the genome is increased, which leads to a greater expression of the genes located in the amplified region, and that this might play an important role in the development of cancer (Mondello et al. 2010).

Additionally, significant increases in the biomarkers of cell death, including KRX, KRL, and CC, and in cytokinesis were found in BN cells. Similar results were obtained in other studies, such as Rohr et al. (2013), León-Mejia et al. (2014), and Anlar et al. (2019). It is to be assumed that when there is an increase in cell death, the organism attempts to meet that need and cell proliferation increases, which results in many cases in these cells becoming defective due to alterations in cell division and signaling proteins (Petsalaki and Zachos 2020). Interestingly, a significant correlation was found between APOP frequency in lymphocytes and KRX in buccal cells, which can indicate that cell death has a systemic effect, regardless of the tissue (Fenech et al. 2011).

The analysis of the characteristics of the groups, it is important to mention that the exposed group is a vulnerable population, of a low socio-economic and educational level, which influenced the life habits and general characteristics found. The results showed a significant correlation between CBMN-Cyt-NBUD and vitamin consumption, between CBMN-Cyt-MN and meat consumption, and between CBMN-Cyt-APOP and meat consumption. In the analysis of the BM-Cyt biomarkers, a significant correlation was found between BM-Cyt-KRL and vitamin consumption and between BM-Cyt-BN and alcohol consumption. As observed, the low consumption of vitamins is an unfavorable factor because many of these compounds are cofactors for proteins involved in the repair of genetic damage (Fenech and Bonassi 2011). Fruits and vegetables are rich in antioxidant cytoprotective components that can counteract the action of ROS and protect cells against oxidant-induced damage (Fenech and Bonassi 2011; Gajski et al. 2018). Furthermore, the general population has a considerable consumption of red and fatty meats. Red meat is an important source of N-nitroso compounds and that these compounds can induce oxidative damage to DNA, increasing the risk of cancer (León-Mejía et al. 2019; Steinberg 2019).

In human biomonitoring, age is a relevant factor in the studied effects of exposed populations. In this study, the age of the control group and the exposed group were similar; however, when the biomarkers in the exposed group were analyzed, a significant correlation was found between the formation of MN in lymphocytes and the appearance of KRL in buccal cells and age. It has been described that DNA damage increases with age as a consequence of unrepaired accumulation of naturally occurring DNA damage, failures in chromosomal segregation and in the cell cycle checkpoint, as well as numerical and structural chromosome aberrations caused by exposure to endogenous, environmental, and occupational genotoxins, to which are added unhealthy lifestyle habits that can increase DNA oxidative damage (Fenech and Bonassi 2011; Gajski et al. 2018). It is also important to mention that the population had unbalanced eating habits, and considerable alcohol consumption. Alcohol consumption can cause DNA damage and is considered a risk factor for cancer (Fenech and Bonassi 2011; Rumgay et al. 2021).

The exposed population of “La Loma” is located around three open-pit coal extraction areas and they live 2 km from the first coal extraction area, a fact that significantly influenced the results of the risk biomarkers analyzed. Coal extraction in this region has notably deteriorated bodies of water, air quality, and soil. Diaz et al. (2013) described the contamination of the soil in the Cesar region with potentially toxic elements such as selenium and zinc, high salinity, and deficiencies of nutritive elements such as phosphorus in the soil. The effects of air pollution on the health of children under 12 years of age have also been described, in which the prevalence of respiratory symptoms and diseases related to exposure to PM_10_ released during coal mining activities was evidenced in the Cesar region (Quiroz et al. 2013). As could be seen in the results, the average exposure time was 20.8 years, a time that shows a high chronic exposure and can be fundamentally associated with the effects found in individuals who live in this locality.

Finally, this study demonstrated that these biomarkers can be used as early predictors of carcinogenesis, and these results contribute to the discussion on the effects of coal mining from the perspective of implementing prevention strategies in populations exposed to pollutants in developing countries.

## Data Availability

All data generated or analyzed during this study are included in this published article (and its supplementary information files).
